# Protective behaviours among orphaned learners in a district of Gauteng province, South Africa

**DOI:** 10.4102/hsag.v30i0.2812

**Published:** 2025-03-17

**Authors:** Thembi V. Simbeni, Mathildah M. Mokgatle

**Affiliations:** 1 Department of Public Health, School of Health Care Sciences, Sefako Makgatho Health Sciences University, Pretoria, South Africa

**Keywords:** resilience, protective behaviours, orphaned adolescents, maternal orphan, double orphan, microsystem

## Abstract

**Background:**

While most orphans have difficulties in coping with the loss of a mother, some of them are motivated by their circumstances to cope with the situation.

**Aim:**

The study aims to explore protective behaviours of maternally orphaned adolescents in their daily immediate environments that enable them to cope with maternal death in Tshwane North District, Gauteng province.

**Setting:**

The setting was secondary schools in Tshwane North of Gauteng province in South Africa.

**Methods:**

A qualitative exploratory design was employed among a subsample of 14 purposively sampled maternally orphaned adolescents using one-on-one, in-depth interviews with open-ended questions. The Ecological Systems Theory was used as a framework for analysis, and data were analysed thematically using NVivo12.

**Results:**

Some of the orphaned adolescents possess protective behaviours, which contribute towards resilience as the adolescents gave an account of their daily lives through their interactions within the microsystem. They engage in meaningful activities to disengage themselves from negative thoughts and unpleasant emotions. Resilient orphans demonstrate hope for the future, maintain a positive self-concept and adopt coping mechanisms such as recalling positive memories of their mother. They actively seek support for their physical and psychological needs, engage in constructive tasks and foster supportive relationships for their psychological well-being.

**Conclusion:**

Possessing personal protective resources and having supportive socioecological resources enable resilience among orphans when facing adversity.

**Contribution:**

Findings of this study will inform interventions geared towards building the resilience of orphaned adolescents to cope with maternal loss.

## Introduction

A global modelling study revealed that between 01 March 2020 and 30 April 2021, 1 134 000 children experienced the loss of one or both parents, caregivers or custodial grandparents from coronavirus disease 2019 (COVID-19) (Lowe et al. 2022). This is approximately one-third of the 3 322 107 fatalities as of April 30 2021 because of COVID-19, showing this enormous challenge of orphanhood (Hillis et al. 2021). In sub-Saharan Africa, over 52 million children have lost one or both parents (Blevins & Kawata 2021). Among other drivers of parental death include diseases at levels endemic to the region, including HIV, malaria, injuries and accidents (Blevins & Kawata 2021). In South Africa, there are over 3 million children who are orphaned (Allman et al. 2023). The UNICEF (United Nations International Children’s Emergency Fund) defines an orphan as any child under the age of 18 years who has lost one or both parents to death. This study assumes the UNICEF definition of an orphan.

The adolescent phase refers to individuals between the ages of 10 years and 19 years (World Health Organization 2021). This phase is characterised as a time of a great change in biological, psychological and social development (Petersen 2021). Parental death impacts the physical, psychological and behavioural states, including the family and school life (Farella Guzzo & Gobbi 2023). Following the loss of a parent, the adolescents aged 12–19 years have been found to be at greater risk as compared to younger children of aged 8 years – 11 years old (Farella Guzzo & Gobbi 2023).

Maternal orphans are children below the age of 18 years who have lost a mother (UNICEF 2014). The adversity of losing a mother results in devastating consequences in the lives of most adolescents (Simbeni & Mokgatle 2023). Many times, the consequence is psychosocial problems that negatively affect the mental health of orphans, their interactions with family members and peers, their performance in school and their engagement in leisure activities with peers (Kaur et al. 2018).

The adversity of parental loss results in the loss of love and care for the orphaned adolescent (Apostol 2024). While other orphaned children have difficulty coping with the loss of a mother, some are motivated by their circumstances to cope with the situation. Resilience has been found to be associated with higher scores on personality measures such as emotional stability, sociability, friendliness, honesty and diligence (Auttama et al. 2021; Garcini et al. 2022; Goodman et al. 2017; Powell et al. 2021; Shiner & Masten 2012). Masten et al. (2003) defined resilience as ‘patterns of positive adaptation in the context of significant risk or adversity’. This definition is applicable in this study.

Studies among vulnerable children reveal that hopefulness, positive prospects for the future and a sense of control over one’s future (self-efficacy) are associated with increased resilience (Betancourt et al. 2011; Du et al. 2017; Garcini et al. 2022; Keshri 2021; Sun, Lam & Chung 2022; Wang et al. 2012). Global studies suggest that caregiving obligations among children prompted personal growth, the need to build resilience and contributed to the child’s ability in supporting their families (Campbell et al. 2012; Rose-Clarke 2022; Skovdal 2010; Zhang et al. 2009). Furthermore, studies have suggested that some orphaned children experience personal growth, emotional maturity and a sense of independence (Diaz-Strong 2022; Little, Akin-Little & Somerville 2011; Murphy, Roberts & Herbeck 2013). Being lively, active and sociable enables children to engage with others and brings about more supportive resources from caregivers and other people outside the immediate family (Masten & Barnes 2018; Shiner & Masten 2012).

There is a paucity of research studies on self-developed protective behaviours of orphaned adolescents in South Africa. In this study, protective behaviours refer to actions that enable and show positive adaptation to the circumstance of having lost a mother as reported by the participants.

In South Africa, there is limited studies on innate behaviours that enable coping with maternal loss. Most studies focus only on orphan experiences and their mental health (Allman et al. 2023; Chilwalo et al. 2024; Khoza & Mokgatle 2021). Research on the protective behaviours of orphans is crucial, as it may lead to interventions that are geared towards instilling internal assets that enable the individual orphaned child to cope with maternal loss.

The Bronfenbrenner’s Ecological Systems Theory was employed as a framework guiding the focus of the study. Bronfenbrenner believed that a child’s development is affected by everything in their surrounding environment. He divided a person’s environment into five different levels: the microsystem, mesosystem, exosystem, macrosystem and chronosystem (Bronfenbrenner 1979). The first construct of the Ecological Systems Theory, the microsystem, informed this study. The microsystem starts with the individual at the centre, and it includes the immediate daily context in which the individual child has direct interaction (Bronfenbrenner 1979). The individual possesses a psychological state, including emotions, inherent internal assets such as a certain perception about the self, coping mechanisms, which influences how he or she perceives and reacts to people and circumstances around him or her (Bronfenbrenner 1979). The immediate family, the school context and relations with the people in it, interaction with peers, extended family members and the neighbourhood are the immediate environments that the child is exposed to and thus the microsystem (Bronfenbrenner 1979). The theory states that a child’s behaviour or interaction with the people in his or her microsystem influences the reciprocal reaction of people towards him or her (Bronfenbrenner 1979). Furthermore, the behaviour of the people within each child’s microsystem influences the child’s reaction towards the people (Bronfenbrenner 1979). Henceforth, the author interpreted that the self-developed protective behaviours of the orphaned adolescents in this study were stimulated by the immediate environment to which the orphans are exposed to. This article presents the self-developed protective behaviours of orphaned adolescents as they emerged within their immediate daily environment that they are exposed to and as influenced by the inherent internal assets possessed by them. This study sought to explore protective behaviours of orphaned adolescents in their daily immediate environments that enable them to cope with maternal death in Tshwane North District, Gauteng province.

## Research methods and design

### Study design and setting

This study adopted a qualitative explorative deductive approach where the researchers developed the interview guide using the theory constructs and propositions and further aligned the presentation of the findings to the theory constructs (Fife & Gossner 2024). The study was conducted in four secondary schools situated in the semi-rural and urban townships of Tshwane North of Gauteng province. These are schools ranked as quintile 1, which means they are non-fee-paying schools. This is because the schools are situated in areas where residents are from economically disadvantaged families, confirmed by the unemployment of most of the caregivers of participants in this study and the reliance on social grants.

### Study population and sample

The study population consisted of non-institutionalised, school-going orphaned adolescents. Both male and female orphaned adolescents, aged 13 to 17 years, who had lost either their mother or both parents, were included in the study. The common characteristic of interest among maternal and double orphans is that they both lost a mother and hence they both met the inclusion criteria. The sample for this study (14) was drawn from the initial larger sample of 25 participants who were recruited as part of the larger mixed-method study (see authors, in press). Selection of the 14 participants from the initial sample size of 25 was based on the data that emerged during analysis; data showing resilience among some of the participants were extracted for presentation in this manuscript. Resilience was shown through the self-reported protective behaviours by the participants (see Table 2 in this article and the participant quotations).

### Data-collection method

We used one-on-one in-depth interviews to collect data. All in-depth interviews were conducted by two people, one researcher and one trained research assistant. Questions were asked in a manner that elicited explanations and descriptions, by asking open-ended questions, probing questions where necessary, by asking for clarification where responses were not clear and by allowing participants to fully respond to the questions asked without interrupting them.

Only one in-depth interview was conducted in a day, and three interviews in a week with a break in between. This allowed the researcher time to listen to the audio-recorded interviews with the co-researcher, to identify shortcomings in the phrasing of certain questions and to rephrase questions or adjust the tool accordingly and subsequently improve the quality of the data, as the data collection continued. Data collection occurred for a period of 3 months.

### Data collection instruments and procedures

An English-language researcher-developed interview guide, which was translated to Setswana, was used to collect the data. The development of the interview guide was informed by Bronfenbrenner’s Ecological Systems Theory. The first part of the tool contained questions on the participants’ sociodemographic data. The questions in the data-collection tool enabled the researcher to gather data on the individual’s self-reports of perceptions about the self, interactions in the home context and school context, peer relations and people in the neighbourhood. Data were collected over 3 months from February to end of April.

Face-to-face, in-depth interviews were used to collect data from the orphaned adolescents, and the first author conducted all the interviews. Data collection took place after school hours in a private room on the school premises to ensure that the environment was quiet and favourable for audio-recording, private and free from interruptions to teaching and learning activities.

The interviews were conducted in Setswana, which is the first language of the participants and a predominant language in the area. Each interview lasted for approximately 45 min. The audio-recorded Setswana data were translated to English. Thematic analysis approach was used to make sense of the data, and common phrases from the data were identified as themes. Themes were used to report the findings.

Questions on the tool were informed by the microsystems constructs of the theory and entailed the individual aspect of the theory constructs that explored emotions, views about the self and future prospects: As a child who has no mother, what comes to your mind when you are alone? Explain what you do when such thoughts come to your mind. Explain what you think about your capabilities. Explain what you think about your behaviour as a child? When you look at your peers in class, explain whether you see a difference or not between you and them. Probe: Tell me more about it, etc. Other questions included questions on the home context and interactions with peers, the school context and relations with family and extended family members.

### Data analysis

The initial bigger study explored the ecological context of orphaned adolescents, which included the microsystem, mesosystem, exosystem and chronosystem. However, in this study, the researcher analysed data on the microsystem. Emerged findings on protective behaviours of maternal adolescent orphans were extracted and presented in this study.

The audio-recorded data were transcribed verbatim. A thematic analysis with a deductive approach, where constructs of the Urie Bronfernbrenner’s Ecological Systems Theory informed the analysis and presentation of findings, was employed (Fife & Gossner 2024). Transcripts were translated into English, proofread and arranged for analysis. The first author and the second author coded the first few transcripts independently and compared their application of codes for intercoder reliability. A codebook was developed and refined over time by the author as a confirmation on the definitions of codes, themes and subthemes. Transcripts were imported into NVivo12, to organise the data and to further apply codes to the transcripts. This approach enabled the researcher to find, analyse and report patterns in the data.

### Trustworthiness

The research assistant was trained in transcribing and translating the data to ensure accurate representation of the participants’ words. Translation and transcription were verified for accuracy by listening to the first three pieces of audio-recorded data and reading the transcribed translated data. Mistakes or misrepresentations of the study participant were shown to the research assistant early during transcription and translation to ensure exact representation of the participant’s words. Peer debriefing sessions were held in the initial phase of data collection for timely identification of weakness and rephrasing of some questions on the tool to allow collection of rich data and to verify the Nvivo data coding and analysis. Prolonged engagement in the field took place, with us interviewing only three participants per week. This was to ensure initiating coding and identifying themes as we collected the data.

### Ethical considerations

Ethical approval of the study was obtained from the Sefako Makgatho Health Sciences University Research Ethics Committee (Ref no. SMUREC/H/241/2017: PG). Written approval to conduct the research at the schools was obtained from the Gauteng Department of Education and from the Tshwane North District Department of Education. The school principals also provided written permission to conduct the study. The prospective participants and their guardians were informed about the aim of the study. Written informed consent was sought from the guardians who lived with the orphaned adolescents. For children who were from child-headed homes and were interested in participating in the study, written consent was sought from the school principals. All the orphaned adolescents gave assent to participate in the study. Informed consent was administered in a language that the participant and the guardians understood. Participants were treated with respect, and participation in this study imposed no psychological or social harm, as the identification and recruitment of the potential study participants was done in a manner that maintained the confidentiality of their personal information. Confidentiality and privacy were ensured. All the participants who participated in interviews were given lunch packs and transport fare, as interviews were conducted after school hours. This gesture was communicated to potential participants who had expressed interest in the study but were concerned about missing their school bus to travel back home because of their participation.

## Results

### Sociodemographic characteristics of the participants

Of the 25 participants in the main study (see Simbeni & Mokgatle 2023), this study is based on the 14 participants who showed resilience following the loss of a mother. Table 1 shows that the ages of all the 14 participants were between 13 years and 17 years. Of all the 14 participants, 10 were females and 4 were males. Of the 14 orphaned adolescents, half of them were maternal orphaned, while the other half were double orphans. Although this study focused on maternal orphans, double orphans met the criteria to be included in this study as they have a characteristic similar to maternal orphans in that they have also lost a mother. They were all living in households within communities. Nine of the participants were living with their grandmothers, three were their siblings and two with their fathers. Only eight of them were receiving a social grant and nine had an unemployed caregiver.

**TABLE 1:** Sociodemographic characteristics of the participants (*N* = 14).

Variables	Number
**Age (years)**	
13–17	14
**Gender**	
Male	4
Female	10
**Who do they live with**	
Grandparents	9
Siblings (sister)	3
Father	2
**Orphan type**	
Maternal orphan	7
Double orphans	7
**Receives a social grant**	
Yes	8
No	6
**Type of a grant**	
Foster care grant	6
Child support grant	3
**Caregiver employed**	
Not getting a grant	5
Yes	5
No	9

**TABLE 2:** Bronfenbrenner’s Ecological Systems Theory constructs of the microsystem and themes aligned to protective behaviours of the maternal orphans in this study.

Theory constructs of the microsystem	Themes Self-reported protective behaviours
The individual orphaned adolescent	Self-engagement in meaningful activities
	Hopeful thoughts about the future
	Positive self-concept
Context of family members, extended family members and neighbours	Actively source support for needs
	Engage in supportive relationships
Peer relations	Interact with peers
The school context	Self-engagement in peer group interaction Self-engagement in constructive tasks Source teacher support

### Findings from the interviews

Data present the findings aligned to the microsystems construct of the Ecological Systems Theory to describe experiences and daily activities of the orphaned adolescents. The focus on the microsystem proves to be the most relevant according to the data in this study. The study therefore drives us to emphasise that strengthening the microsystem can facilitate self-development and resilience in the context of low- and middle-income countries (LMICs) where there are limited resources for investing in high-level interventions. The narratives from the face-to-face interviews showed that some of the adolescent orphans revealed protective behaviours that portray resilience. The following themes describe protective behaviours of the orphaned adolescents in coping with maternal death such as self-engagement in meaningful activities, being hopeful about the future, having a positive self-concept, active source of support for needs and engaging in supportive relationships and social interaction, self-engagement in constructive tasks, self-engagement in peer group interaction and sourcing teacher support.

## The individual orphaned adolescent

### Self-engagement in meaningful activities

Participants reported that they engage themselves in activities whenever they experience thoughts of wanting to end their lives, when they miss their mother or when they are sad about something. This was done to distract themselves from their unpleasant thoughts and emotions and, for some, it was to help them forget about their unpleasant situation at home. The participants used writing, music and mental activities such as playing chess, practising mathematics and positive thinking about their mother as a way of coping. When a participant was asked to explain what she does when she is bothered by something when she is at school she stated:

‘Most of the time I sing at school like I only sing during break time, we sing as girls from my class about four or five or us. Mostly singing does remove a lot of things from your mind.’ (17 years old, female, maternal orphan)

‘It crosses my mind sometimes, but I keep myself busy with something maybe I just write a song lyric or I just write a poem. From there I forget everything, yes actually that’s how I handle things.’ (16 years old, male, double orphan)

A participant who is heading a home said:

‘When I practice maths eish, even when I play chess because it keeps my mind too busy, and I forget all the things happening in my life.’ (17 years old, male, maternal orphan)

A participant explained what she and her sibling do when missing their mother:

‘My little sibling and I came up with the idea that before we sleep, we should just imagine things that are not there. So, I also came up with the idea that ok since we are able to think about non-existent things, let us think about the good things that our mother used to do for us the time when we were still young. So, I started doing this thing, every time when I go to bed I first think about her then I am able to sleep. I think about things that she used to do for me, sometimes we would just be sitting as a family sharing jokes and laughing so that I can just feel that love she had for me.’ (14 years old, female, maternal orphan)

### Hopeful thoughts about the future

Furthermore, participants were hopeful about the future, and to some participants, hopefulness about the future gave them some consolation in their circumstance of having lost a mother:

‘The thing that comes to my mind … I always console myself by telling myself that I’m going to succeed as people always say she is no more, she has passed on, she is resting so. What comes to my mind is that I succeed in my studies.’ (17 years old, female, maternal orphan)

When a participant was asked to explain how she foresees her future as a child who has lost a mother she said:

‘I see myself succeeding in my education, I see myself being a good example to my younger brother, I see myself making my grandmother happy, I see myself making my mother happy wherever she is, I see myself growing spiritually … yes, I see myself succeeding in every way.’ (14 years old, female, maternal orphan)

A participant who is heading a home, when asked to explain how he foresees his future as a child who has lost both parents he stated:

‘I want to be the best IT [*Information Technologist*] in the world, I want to open my company that deals with creating Apps, programmes, software, things like that, I see a bright future for myself, I see myself fulfilling my dreams.’ (17 years old, male, double orphan)

### Positive self-concept

The orphaned adolescents had good self-esteem, which was revealed in their positive explanation of their behaviours, their self-image and ability to communicate their views, when a participant was asked to explain how he views his behaviour as compared to peers in his class he stated:

‘I think I am okay, I take the whole 100% when it comes to behaviour, I don’t compare myself with anyone.’ (17 years old, male, double orphan)

‘I think I am a good person, who is righteous, I don’t have issues with anyone. Talk about my physical appearance I think I am fine and nothing wrong on me.’ (17 years old, female, maternal orphan)

## The immediate daily context of family members, extended family members and the neighbours

### Actively source support for needs

This study’s findings show that participants sourced support for their physical and psychological needs from friends and adults in their lives. The need for food was mainly sourced from peers who lived closer to them at home when there was no food at home and from close friends. Also, they borrowed and exchanged clothes with their female friends. Narratives also show that participants sourced support for psychological needs by telling someone about things that bothered them. A participant living with her eldest sibling who is unemployed stated:

‘No, that’s Itumeleng, Pholoso lives next door to my home, she’s the one I go to when there is no food at night. I go to her and ask her to at least dish a little for us because Itumeleng lives a bit far from my home.’ (17 years old, female, maternal orphan)

When asked to explain what he does when he is home and bothered by something:

‘I tell my brother who is not living with us, in a way I feel like I am relieving stress because I tell him everything that hurts me. He would also tell me that he doesn’t want anything bad happening to me.’ (17 years old, male, maternal orphan)

‘Sometimes when I go to my sister, I would feel helpless and useless then after talking to her that’s when I express the pain that I thought things were things were like this only to find that they are someway, then I start feeling useful.’ (16 years old, female, double orphan)

### Engage in supportive relationships

Orphans in this study preferred forming and maintaining friendships with consistent people who knew their situation, understood them and therefore would not talk a lot about parents or in a way that would hurt them:

‘My friend supports me, she is more like a sister to me. She supports me in every way, even when I must go to the clinic she asks “when is your next appointment” then she accompanies me, she is more supportive than my family.’ (17 years old, female, double orphan)

‘I have friends that I have been with since primary then we went to the same secondary so I prefer to be with them because they know that there are some things, they don’t say to me, they know those things will hurt me, like they know me, so I prefer to be with them.’ (14 years old, female, maternal orphan)

A participant who has lost both parents, stated that she opens up to her sister when worried about something at home and that she gets assistance from the person she is opening up to caregiver’s support:

‘I get satisfied. Sometimes when I go to her, I feel helpless and useless. Then after talking to her, that’s when I express the pain that I thought things were like this only to find that they are some other way. Then I start feeling useful.’ (17 years old, female, double orphan)

## Peer relations

Some orphans in this study acknowledged the benefit of spending time with peers and interacting with them. They reported that spending time with peers is enjoyable, in that playing, talking and laughing with friends help them to ‘*forget a bit*’ including to not miss the mother. Some of these social interactions are a source of support where each can share their experienced problems with close peers:

‘What makes me happy is that they are open with me, they tell me when they have problems and I also tell them when I have problems and sometimes, we play games with the kids on my street, or we make jokes because they love jokes.’ (16 years old, female, double orphan)

A girl who has lost her mother and is living with her sister stated:

‘My friends are the kind of friends that support me. They give me strength and they are fine. When I’m with them, it feels like they are my siblings.’ (17 years old, female, maternal orphan)

## The school context

### Self-engagement in peer group interaction

The narratives show that orphans in this study acknowledged that interacting with peers at school was beneficial to them. They reported that freely talking to peers and making jokes brought forth fun, laughter and ‘relief’:

‘Mostly I love being in groups at school where we talk about life and then we will have fun, making jokes, I like sitting in groups because I know I will laugh the most.’ (17 years old, female, maternal orphan)

When a participant was asked to explain what makes her happy at school, she said:

‘When I am at school that thing of my aunt not doing anything for me is no longer there, I focus on the other kids at school that don’t criticise me, so we laugh and make jokes and the class becomes nice.’ (17 years old, female, maternal orphan)

‘I am able to talk to a lot of children, I feel relieved when I am at school.’ (17 years old, female, maternal orphan)

### Self-engagement engagement in constructive tasks

Some participants said that being engaged in a favourite school subject and doing well in it made them happy at school. Practising mathematical sums, playing chess and competing for marks in school subjects were reported as things that kept the mind busy and helped to forget about the things that are happening in life. Also, orphans in this study reported that these activities helped in preventing from thinking a lot, a participant in grade 11 who has lost both parents, stated what made him happy at school:

‘When I practice math, Eish! Even when I play chess, because it makes me concentrate a lot and I forget all the things happening in my life.’ (17 years old, male, double orphan)

‘When a teacher teaches something that makes me not think too much that’s when I become happy. When we are busy with my favourite subject and I perform well in it, that makes me happy.’ (17 years old, male, double orphan)

### Source teacher support

Some reported that some teachers were a source of support, encouraging them to continue with their schooling during and after pregnancy. Some teachers had supported orphans in this study when dealing with the loss of a mother and gave the participants a shoulder to cry on, care and advice when needed. Some orphans in this study also mentioned that they could talk to specific supportive teachers almost every day when they needed. A teenage mother who has lost her mother, when asked about how her teachers were treating her at school she stated:

‘This other time when I was pregnant, she was comforting me. She is the one who gave me courage to go to school. That even though I am pregnant I should go back to school and continue with my studies. She was the person who was supporting me at school, the one I could talk to almost every day.’ (17 years old, female, maternal orphan)

A girl in grade 10 who has lost her mother, when asked about how the teachers at school treated them, stated:

‘She is very attentive. She would give me her attention. That was the first thing I loved about her. She would give me a shoulder to cry on and she would give me advice based on what she was thinking. So that’s what she used to do.’ (14 years old, female, maternal orphan)

## Discussion

This study explored the protective behaviours of non-institutionalised orphaned adolescents attending high schools in both semi-rural areas and urban townships of the Tshwane North District in South Africa. The participants were living in families within their communities, cared for by grandmothers, aunt or uncles, and very few were living with their fathers and only two of them lived with siblings heading their households.

Results in this study showed that participants had a positive view of themselves and believed in their abilities. Having a good sense of self-worth, and coping self-ability were associated with increased resilience in studies conducted elsewhere (Betancourt et al. 2011; Li et al. 2020). Participants in this study were hopeful about the future, and expecting success in the future gave them some comfort and courage in their current situation. This study’s results are similar to findings in other studies that revealed that hopefulness, positive prospects about the future, a sense of control over one’s future and self-efficacy were associated with increased resilience (Betancourt et al. 2011; Du et al. 2017; Garcini et al. 2022; Keshri 2021; Sun et al. 2022; Wang et al. 2012). Human beings are driven by different reward systems to adjust to the situation, and people who believe that they will be successful tend to persist longer when faced with challenges (Bandura & Walters 1977; Masten 2011; Oshri et al. 2018; Sun et al. 2022).

This study found that because the participant and her sibling started thinking about previous positive experiences shared with the mother who had died, they were able to ‘feel the love that their mother used to give them, to laugh and to sleep better’. This new phenomenon was also suggested as one of the family resilience enhancing strategies in helping family members to move beyond bereavement; this is done by reconnecting with the past by sharing sweet memories of the lost loved one (Walsh 2020).

Study participants engaged themselves in activities to distract themselves from sadness, suicidal ideation and distress associated with their circumstance of having lost parents. Self-engagement in writing, music and mental activities distracts themselves from unpleasant thoughts and emotions. Distracting oneself by keeping busy, socialising and doing something good for oneself is one of the ways that is reported to be helpful in reducing suicidal thoughts (Stanley et al. 2021; Ugboha, Nwokocha & Barnabas 2022).

An individual has physical, emotional and psychological states that influence how they respond to their environment (Bronfenbrenner 1979). Good self-concept, having positive prospects, practising positive thinking, self-sustenance income and self-engagement in activities to distract themselves from suicidal ideation and other unpleasant thoughts are protective internal assets that were present within individual participants in this study as shown by the narratives. These internal assets enabled adaptive coping and resilience to the circumstance of having lost a mother.

This study also found that participants actively sourced support for their physical and psychological needs by communicating their needs and talking about things that bothered them to people in their context. They also formed and maintained friendships where support was mutual and protective of their emotional state. Participants were selective in the kind of peers that they interact with to protect themselves from being hurt. They engaged in group interactions that make them laugh and distract them from worries emanating from home. Acknowledging one’s own psychological needs and sourcing support from people is an indication of the motivation to positively adapt to the situation. These findings are consistent with the suggestion that being lively, active and sociable enables children to engage with others and bring about more supportive resources from the caregiver and from people outside family context (Powell et al. 2021; Shiner & Masten 2012).

We also found that because participants in this study socially interacted with peers at school and at home, they were able to source emotional support, to experience feelings of enjoyment and relief from distress. These findings are consistent with a number of studies that have shown that having good quality peer relations is one of the protective factors associated with mental health resilience among orphaned adolescents (Betancourt et al. 2011; Collishaw et al. 2016; Li et al. 2015; Mishra & Sondhi 2022). Urie Bronfenbrenner’s Ecological Systems Theory (1979) states that relationships in the microsystem are bidirectional. This means that an individual’s reaction to the people in his or her microsystem will affect how they treat the individual in return (Bronfenbrenner 1979). Individual participants in this study were able to relate positively with people and communicate their needs and concerns effectively. As a result, they were able to draw emotional and social support from people in daily contexts and to adapt positively to the situation of having lost a mother.

## Recommendations

The school system needs to integrate programmes geared towards building every child’s self-concept and the motivation to master situations. This can be done through school-based and community-based skills training, as well as group-based tasks that involve problem-solving and decision-making. Learning new skills where there is mastery may improve the self-esteem, a positive sense of self-worth and the motivation to take control of situations. The suggested skills training may also support orphans in this regard.

Schools need to provide a safe and supportive environment that identifies and supports vulnerable learners holistically. This can be achieved through collaborative work between the child’s guardian, social development department through community social workers and the school system to support orphaned children and adolescents. Guardians need to be empowered to support orphaned children and adolescents in school-related matters, as well as on how to support them emotionally. This can be done through community-based social workers at local Non Governmental Organisations (NGOs) or by the school system.

To support orphans who are not able to initiate interaction with peers, whole-school organised playtime group activities, which should induce the interaction of orphaned children with their peers, are recommended. The interaction would facilitate the establishment of friendships.

A participant in this study reported the use of a coping mechanism that involved thinking about previous positive experiences shared with the mother who has died, to ‘feel the love that their mother used to give them, to laugh and to sleep better’. This study has contributed to new knowledge in this regard; this coping mechanism can be explored further in future research to establish its efficacy in assisting orphans to cope with maternal loss.

## Conclusion

Some orphans adapt positively to the circumstance of maternal death. In this study, participants who showed resilience to the circumstance of maternal loss had a positive self-concept, an internal assert that formed the basis in enabling the forming and maintaining of friendships with peers, communicating own views and sourcing support for their physical and psychological needs. Good self-concept is a prerequisite for one to see potential in oneself, expect success and take control of situations. Participants in this study disengaged from negative thoughts and unpleasant emotions by engaging themselves in meaningful activities. Participants in this study benefited psychologically from socially interacting with peers at school and at home. Close friendships with peers were a context where peers shared their challenging experiences and where support is mutual. In this study, participants who were expecting future success showed resilience to their circumstances of maternal loss. Schooling gives children expectations for their future and the resources to realise their goals.
